# Changing population characteristics, effect-measure modification, and cancer risk factor identification

**DOI:** 10.1186/1742-5573-4-10

**Published:** 2007-10-01

**Authors:** Martha L Slattery, Maureen A Murtaugh, Charles Quesenberry, Bette J Caan, Sandra Edwards, Carol Sweeney

**Affiliations:** 1 University of Utah, School of Medicine, Department of Medicine, Salt Lake City, Utah 84108 USA; 2 Kaiser Permanente Medical Research Program, Department of Research, 3505 Broadway, Oakland CA 94611 USA

## Abstract

Epidemiologic studies have identified a number of lifestyle factors, e.g. diet, obesity, and use of certain medications, which affect risk of colon cancer. However, the magnitude and significance of risk factor-disease associations differ among studies. We propose that population trends of changing prevalence of risk factors explains some of the variability between studies when factors that change prevalence also modify the effect of other risk factors. We used data collected from population-based control who were selected as study participants for two time periods, 1991–1994 and 1997–2000, along with data from the literature, to examine changes in the population prevalence of aspirin and non-steroidal anti-inflammatory medication (NSAID) use, obesity, and hormone replacement therapy (HRT) over time. Data from a population-based colon cancer case-control study were used to estimate effect-measurement modification among these factors. Sizeable changes in aspirin use, HRT use, and the proportion of the population that is obese were observed between the 1980s and 2000. Use of NSAIDs interacted with BMI and HRT; HRT use interacted with body mass index (BMI). We estimate that as the prevalence of NSAIDs use changed from 10% to almost 50%, the colon cancer relative risk associated with BMI >30 would change from 1.3 to 1.9 because of the modifying effect of NSAIDs. Similarly, the relative risk estimated for BMI would increase as the prevalence of use of HRT among post-menopausal women increased. In conclusion, as population characteristics change over time, these changes may have an influence on relative risk estimates for colon cancer for other exposures because of effect-measure modification. The impact of population changes on comparability between epidemiologic studies can be kept to a minimum if investigators assess exposure-disease associations within strata of other exposures, and present results in a manner that allows comparisons across studies. Effect-measure modification is an important component of data analysis that should be evaluated to obtain a complete understanding of disease etiology.

## Background

Epidemiologic investigations have detected associations between colon cancer and several diet and lifestyle risk factors, but the relative importance of risk factors has changed over time [1,2]. Studies of diet and colon cancer conducted in the early 1980s showed that fat was statistically significantly associated with colon cancer, but more recent studies have not supported that association [3-6]. More recently use of aspirin and hormone replacement therapy (HRT) have emerged as being statistically significant risk factors for colon cancer as well as significantly interacting with other risk factors [7-9]. For example, it has been shown that the association between obesity as indicated by a high body mass index (BMI) and increased relative risk of colon cancer is influenced by estrogen or hormone replacement therapy use [10,11]; reports suggest that aspirin may influence the colon cancer relative risk associated with fat intake [12,13]; other data support the role of effect modification on diet and lifestyle factors in colon cancer etiology [12,14].

Inconsistencies in results for risk factor-disease associations for colon cancer have been attributed to a variety of non-biological factors. Since earlier results for colon cancer came primarily from case-control studies, while cohort studies contributed more to the recent literature, study design, i.e. the potential biased reporting in case-control studies, has been cited as a potential explanation for differences between results of older and more recent studies[15]. However, results of cohort studies may be affected by measurement error from imprecise exposure assessment when data are collected using a self-administered format. Thus case-control and cohort studies each have strengths and weaknesses, yet many risk factor associations are observed to be similar from both case-control and cohort studies. While random and systematic errors may explain differences in study results, even for similar, well-conducted studies, a very plausible explanation for a trend in study results is a change in prevalence of exposures that modify effects of other risk factors.

We propose that risk factors associated with colon cancer at the population level exist in a shifting context. We hypothesize that the ability of studies to estimate the influence of risk factors for colon cancer depends on the presence of effect-measure modifiers that are changing in the population. We hypothesize that change in the estimated relative risk from a given exposure can occur if prevalence of other exposures that modify its risk are changing. Thus, it is possible that differences in risk factors identified between studies can be attributed, at least in part, to differences in the prevalence of important population characteristics.

Changes in U.S. population during the last quarter of the 20th century in diet, physical activity, and weight, characteristics that affect colon cancer risk, have been described based on national surveys [16]. In this paper we examine changes in the use of aspirin, HRT, and obesity in population-based samples from two western states between the early 1990s and the late 1990s. Data from these populations were rigorously collected using the same questionnaire and recruitment methods. We evaluate trends in use of HRT and aspirin over the last three decades of the 20th century as reported in the literature and in National Health and Nutrition Examination Survey (NHANES) data.

To test our hypothesis that effect-measure modifiers can influence population level risk of other factors, we consider the inter-relationships of aspirin and other NSAID use, BMI, and HRT in altering the risk of colon cancer. We focus particularly on these factors since their prevalence has changed dramatically over the past decades and they appear to play a major role in modulating colon cancer risk factors.

## Methods

### Study Population

Data from two case-control study populations are included in these analyses[17,18]: a population-based case-control study of colon cancer of cases and controls selected between 1991 and 1994 and a population-based case-control study of rectal cancer where cases and controls were identified between 1997 and 2000. Both studies used identical methods to identify, recruit, and interview study participants. In both studies, participants were asked to recall exposures two years prior to the date of selection for the study. Methods for both studies have been described in the published literature and are summarized below[19,20]. The colon cancer study data were used to estimate associations, including effect-measure modification or interaction between exposures. Controls from both cases-control studies were used to estimate changes in exposures over time. Participants in both studies were from the Kaiser Permanente Medical Care Program of Northern California (KPMCP) and the state of Utah. Response rates for controls from the two studies were comparable.

The colon cancer study included cases with a first primary of colon cancer (International Classification of Diseases of Oncology, 2nd edition codes 18.0, 18.2 to 18.9) diagnosed between October 1, 1991 and September 30, 1994. The rectal cancer study included cases with a first primary tumor in the rectosigmoid junction or rectum diagnosed between May 1997 and May 2001. For both studies, controls were frequency-matched on sex and 5-year age group to the cases. Case eligibility was determined by the Surveillance Epidemiology and End Results (SEER) Cancer Registries in Northern California and in Utah and identified using a rapid-reporting system. At the KPMCP, controls were randomly selected from membership lists. In Utah controls 65 years and older were randomly selected from Health Care Finance Administration (HCFA) lists and controls younger than 65 were randomly selected from driver's license lists. To be eligible for the study, participants had to be between 30 and 79 years of age at time of diagnosis, English speaking, mentally competent to complete the interview, and could not have had previous colorectal cancer. Cases with known (as indicated on the pathology report) familial adenomatous polyposis, ulcerative colitis, or Crohn's disease were excluded.

A total of 1346 colon cancer cases and 1544 matched controls were interviewed between January 1991 and December 1994 and 952 cases and 1205 controls were interviewed between October 1997 and January 2002 as part of the rectal cancer study and are included in these analyses. Of participants contacted between 1991 and 1994, 80.8% of cases and 71.8% of controls participated; between 1997 and 2001, 68.8% of controls participated.

### Data Collection

Data were collected by trained and certified interviewers using laptop computers. For both the colon and rectal cancer studies, data were collected in an identical manner, using the same study questionnaire and the same quality control procedures [19-21]. The interview took approximately two hours. Quality control methods were used in both studies to assure that interview procedures were implemented consistently and have been described in detail [19,20].

For both studies, dietary intake was ascertained using an adaptation of the CARDIA diet history [20,22,23]. Participants were asked to recall foods eaten, the frequency which they were eaten, serving size, and if fats were added in the preparation. Nutrient information was obtained by converting food intake data into nutrient data using the Minnesota Nutrition Coding Center (NCC) nutrient database. Height and weight were measured at the time of interview and weight also was reported for the two and five years prior to interview. The body mass index (BMI) of kg/m^2 ^was calculated for men and women. Other information of relevance to this analysis included reproductive history, use of aspirin and NSAIDs on a regular basis, and physical activity [24]. The interview included questions for women on the use of exogenous hormones such as estrogen, progestin, or other female hormones for both contraceptive and non-contraceptive purposes. Dates of first and last use and duration of use of HRT were ascertained. Menopause history including reason for menopause, natural or surgery, and age at menopause, was obtained.

### Statistical Methods

To evaluate differences in population characteristics for the two time periods (i.e. 1991–1994 and 1997–2001), we compare exposure prevalence among controls after adjusting for possible age and sex differences. Variables assessed included variables thought to be important effect-measure modifiers of other variables. These included, BMI, physical activity, NSAID use (includes use of aspirin), and HRT use among post-menopausal women.

In order to describe population changes in exposures to these factors over a longer time period, we used results from national surveys conducted in the U.S., and the published literature, to obtain estimates of exposure prevalence for obesity and NSAID use in the 1970s and 1980s. BMI for men and women was available from the NHANES surveys [25]. We could not find suitable national survey data to estimate changes in the prevalence of aspirin use. Although NHANES surveys have asked about aspirin use, NHANES surveys over this time period have used questions with different definitions of aspirin use, and different groupings of aspirin, acetominophen, and other NSAIDs, so that the data are not comparable over time. We therefore estimated prevalence of NSAID use in the 1970s and 1980s as reported from other population-based studies that used questions similar to those in our own studies [26,27].

For colon cancer relative risk associations, reported weight for the period two years prior to diagnosis was used to calculate BMI. Using aspirin or non-steroidal anti-inflammatory drugs on a regular basis (defined as at least three times per week for one month) within two years prior to diagnosis was considered positive use, while those not using aspirin or non-steroidal anti-inflammatory drugs during this time period were considered non-users. HRT use was defined as having used HRT within the past two years. Data were categorized into groups based on respondent response to using aspirin/NSAIDs, HRT, and BMI cut-points of 25 and 30. For physical activity levels and western diet, categories were based on distribution of these continuous variables as reported for the colon cancer study.

To provide an example of the potential for risk estimates to change due to change in the prevalence of effect-measure modifiers, we estimated odds ratios for the relative risk of colon cancer associated with obesity (BMI ≥ 30) compared to individuals whose BMI was ≤ 23 stratified by HRT users and non-users and by NSAID/aspirin users and nonusers. Unconditional logistic regression models were used to estimate odds ratios associated with effect-modification adjusting for confounding factors that included energy intake, physical activity level, dietary calcium and fiber, HRT (NSAID/aspirin model), and NSAIDs/aspirin (HRT use only model). Statistical testing for interaction was determined by the relative excess risk from interaction (RERI) [28,29]. The RERI was used to estimate the relative strength of the interactive effect and can be interpreted as the excess risk due to interaction relative to the risk without exposure. Using this model risk estimates are estimated for exposures with and without the presence of other exposures and it is estimated if the difference between the combined exposures are greater or less than would be expected when the risk from the two independent exposures are added. Statistical analyses were done using SAS statistical software (Cary, NC).

To illustrate the impact of changes in prevalence of use of aspirin and HRT over time on risk estimates for other risk factors, we estimated ORs for BMI (highest quartile vs. lowest) in hypothetical populations with different prevalence of NSAID/aspirin and HRT use. To develop these estimates we used the ORs for obesity or a BMI of 30 or more relative to those with a BMI of 23 or less that were observed in our study population. These risk estimates were calculated within strata of NSAID/aspirin users (risk estimate used was 2.5 from the stratified model) and non-users (risk estimate of 1.2 used from stratified model) and HRT users (risk estimate of 2.1 used for strata of users) and non-users (risk estimate of 0.8 used for strata of non-users). We assumed that risk estimates for BMI within these circumstances would be constant, although variation in exposure to NSAID/aspirin and HRT use in the population would influence the actual BMI detected risk. We calculated the population risk as the variable exposures of NSAIDS/aspirin and HRT using population distributions of 5% users, 95% non-users, then a population with 10% aspirin users, 90% non-users, etc. Additional File 1 shows the calculation for these estimates. These predicted population ORs are shown over a range of percentages of use of NSAIDs and HRT that are comparable to changes reported in these exposures over time.

## Results

**Figure 1:**
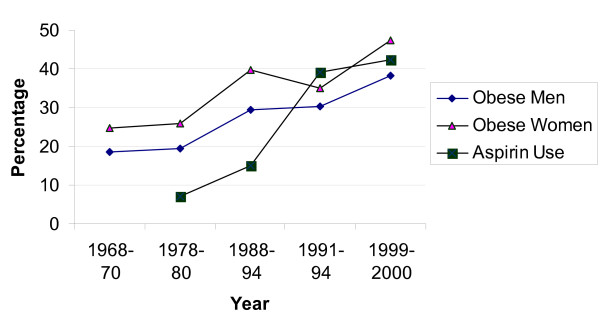
Changes in obesity and aspirin use between 1970 and 2000 in U. S. populations. Obesity data prior to 1991 from NHANES [25], for 1991–94 from colon cancer case-control study [17], and for 1999–2000 from rectal cancer study [42]. Aspirin data from 1970s and 1980 from references [26, 27] and for 1991–94 from colon cancer case-control study [17], and for 1999–2000 from rectal cancer study [42].

Figure 1 illustrates changes in obesity and aspirin use over the last two decades. Prevalence of obesity comes from NHANES data and data from our two control populations. In a 30 year time period, the prevalence of obesity has doubled. The prevalence of aspirin use dramatically increased between population-based studies conducted in the late 1970s and early 1980s, in which use was reported at 7% [26] and 15% [27] respectively, and one conducted in 1997–2000, in which use was over 40%. Our data demonstrate the dramatic change in reported use of HRT, increasing from 35.6% of post-menopausal women in 1991–1994 to 50.7% in 1997–2000 (Table 1). We further evaluated the joint distribution of risk factors over time, using data from the control subjects (Tables 2 and 3).

**Table 1:** BMI (kg/m^2^), HRT use, and current NSAID/aspirin use reported during 1991–1994 and 1997–2001 by two population-based control groups^1^

		1991–1994	1997–2000	p value^2^
		N (%)^3^	N (%)	
NSAIDs/aspirin:	No	938 (60.8)	692 (57.6)	0.02
	Yes	605 (39.2)	510 (42.4)	
				
HRT use:	No	364 (64.4)	188 (49.3)	<0.01
	Yes	201 (35.6)	193 (50.7)	
				
BMI^1^	≤ 23	287 (18.8)	173 (14.5)	<0.01
	24–25	272 (17.8)	174 (14.6)	
	26–29	507 (33.2)	427 (35.9)	
	≥ 30	461 (30.2)	417 (35.0)	

^1^Data from controls participating in population-based studies of colon and rectal cancer in Utah and Northern California [18]

^2^p values based on χ^2 ^statistic from contingency tables with Mantel-Haenszel adjustment.

^3^Numbers vary slightly because of missing data for the various variables; HRT use is limited to data from post-menopausal women.

**Table 2:** BMI and current NSAID/aspirin use stratified by HRT users and non-users^1 ^in 1991–1994 and 1997–2001 [17, 18]

	1991–1994		1997–2001		
	HRT Yes (36% of population) N (%)	HRT No (648% of population) N (%)	HRT Yes (51% of population) N (%)	HRT No (49% of population) N (%)	P value^2^

BMI (kg/m^2^)					
≤ 23	49 (25.0)	80 (22.4)	41 (21.9)	32 (17.2)	<0.01
24–25	36 (18.4)	59 (16.5)	28 (15.0)	30 (16.1)	
26–29	56 (28.6)	98 (27.5)	64 (34.2)	55 (29.6)	
≥ 30	55 (28.1)	120 (33.6)	54 (28.9)	69 (37.1)	
					
NSAIDs/aspirin					
Yes	91 (45.3)	137 (37.7)	93 (48.2)	85 (45.7)	<0.01
No	110 (54.7)	226 (62.3)	100 (51.8)	101 (54.3)	

^1^Restricted to post-menopausal women

^2^p value compares differences in distribution over time using a χ^2 ^statistic.

**Table 3:** BMI among current NSAID/aspirin users and non-users in 1991–1994 and in 1997–2001 [17, 18]

	1991–1994		1997–2001		
	NSAIDs/aspirin Yes (39% of population) N (%)	NSAIDs/aspirin No (61% of population) N (%)	NSAIDs/aspirin Yes (42% of population) N (%)	NSAIDs/aspirin No (58% of population) N (%)	P value^1^

BMI (kg/m^2^)					
≤ 23	113 (19.0)	174 (18.7)	63 (12.5)	109 (16.0)	0.65
24–25	104 (17.5)	168 (18.1)	73 (14.5)	99 (14.5)	
26–29	200 (33.6)	306 (32.9)	181 (35.8)	246 (36.0)	
≥ 30	178 (29.9)	283 (30.4)	188 (37.2)	229 (33.5)	

^1^p value compares differences in distribution over time using a χ^2 ^statistic.

We detected a significant interaction between NSAID use and HRT on colon cancer relative risk (RERI p value 0.06) (Table 4). The interaction is such that the only observed increased in colon cancer relative risk from (OR 2.0 95% CI 1.3–2.9) is among women who use neither NSAIDs nor HRT. Not using HRT did not result in an increased relative risk of colon cancer among women who used NSAIDs on a regular basis.

**Table 4:** Relative risk of colon cancer associated with NSAID/aspirin and HRT use, and their interaction, among post-menopausal women only, 1991–1994

		All post-menopausal women		NSAID/aspirin Use Stratified			
		
		Cases/Controls (N)	Adjusted	Case/Control (N)		Yes	No
		
			OR (95% CI)	Yes	No	OR^1 ^(95% CI)	OR^1 ^(95% CI)
HRT use	Yes	138/198	1.0	52/89	86/109	1.0	1.3 (0.8–2.0)
	No	344/361	1.4 (1.0–1.8)	90/136	254/225	1.1 (0.7–1.7)	2.0 (1.3–2.9)
RERI^2 ^(95% CI);p value							(-0.03, 1.21);0.06

^1^Adjusted for age, energy intake, physical activity level, BMI, dietary calcium and fiber and NSAIDs/aspirin.

^2^Relative excess risk for interaction, a measure of the amount of effect-measure modification on the additive scale. A value of zero would represent no difference in ORs.

Odds ratios for associations between BMI and colon cancer relative risk differed significantly between subjects who did and did not use NSAIDS and HRT (Table 5). Among those who used NSAIDs on a regular basis, obesity significantly increased relative risk of colon cancer while among non-users there was little increase in risk associated with being obese. Similarly for HRT users, there was a significant increase relative risk associated with obesity while among post-menopausal women who did not use HRT, there was no increased relative risk associated with obesity.

**Table 5:** Colon cancer relative risk associated with BMI (kg/m^2^), by NSAID/aspirin and HRT use,1991–1994

NSAIDS/Aspirin		Everyone	Adjusted	Yes	No	Yes	No
		Case/Control N	OR (95% CI)^1^	Case/Control N	Case/Control N	OR (95% CI)	

BMI	≤ 23	202/307	1.0	41/115	161/192	1.0	2.4 (1.6,3.6)
	24–25	233/296	1.2 (0.9–1.5)	71/120	162/176	1.6 (1.02,2.6)	2.5 (1.6,3.8)
	26–29	459/528	1.3 (1.0–1.6)	136/189	323/339	1.9 (1.3,3.0)	2.6 (1.7,3.8)
	≥ 30	443/404	1.6 (1.2–2.0)	179/177	264/227	2.7 (1.8,4.1)	3.0 (2.0,4.5)
RERI^2^(95% CI); p value						-1.09 (-2.30, 0.11); 0.07	
							
HRT^3^		Everyone	Adjusted	Yes	No	Yes	No

		Case/Control N	OR (95% CI)^4^	Case/Control N	Case/Control N	OR (95% CI)	OR (95% CI)

BMI	≤ 23	109/145	1.0	27/64	82/81	1.0	2.6 (1.5,4.6)
	24–25	97/109	1.2 (0.8–1.7)	39/44	58/66	2.1 (1.1,4.0)	2.3 (1.3,4.1)
	26–29	139/151	1.2 (0.9–1.7)	35/49	105/102	1.7 (0.9,3.3)	2.6 (1.5,4.5)
	≥ 30	137/154	1.1 (0.8–1.5)	37/41	100/113	2.1 (1.1,4.0)	2.1 (1.2,3.5)
RERI^4 ^(95% CI); p value						-1.68 (-3.53,0.17); 0.07	

^1^Adjusted for age, sex, energy intake, physical activity level, dietary calcium and fiber, NSAIDs/aspirin

^2^Relative excess risk from interaction, a measure of the amount of effect-measure modification on the additive scale. A value of zero would represent no difference in ORs.

^3^Analysis restricted to post-menopausal women

^4^Adjusted for age, sex, energy intake, physical activity level, dietary calcium and fiber, NSAIDs/aspirin, and HRT; restricted to post-menopausal women

**Figure 2:**
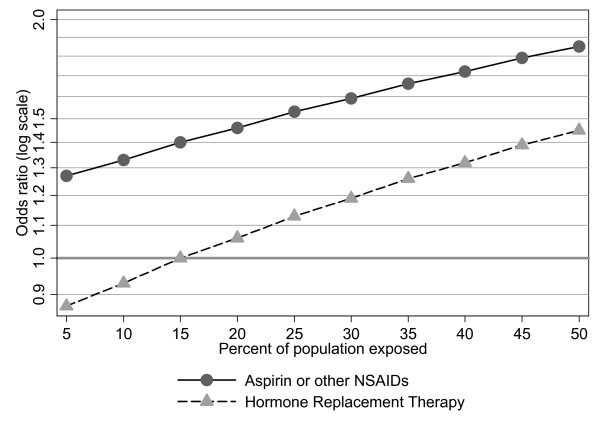
Predicted ORs for colon cancer associated with obesity (BMI ≥ 30 kg/m^2 ^versus lowest quartile, BMI ≤ 23 kg/m^2^) where obesity is held constant but with varying prevalence of NSAID or HRT use. ORs calculated from multiple logistic regression models for stratum-specific ORs for obesity in users and non-users of aspirin, and users and non-users of HRT among post-menopausal women. Adjusted for age, sex (NSAIDS/aspirin use only), energy intake, physical activity level, dietary calcium and fiber, NSAIDs/aspirin (HRT model only).

Figure 2 illustrates the relative risk associated with obesity by various levels of exposure to NSAIDs and HRT. We estimate that for a population in which 5% use aspirin or NSAIDs, the overall OR for obesity (BMI of 30 or more) that would be detected would be less than 1.3, while the OR that would be detected at the population level would increase to over 1.8 in a population with 50% NSAIDs users. Similarly, the OR for obesity among women in a population with few HRT users that would be detected would be close to null, but would increase to 1.45 among women if the prevalence of HRT use was 50%.

## Conclusion

We have demonstrated using data from our studies and the literature that BMI, HRT use, and aspirin use have changed over time. In addition to documenting these changes, we have shown, through assessment of interaction, the potential impact of those changes on ORs for colon cancer relative risk estimates in the population. The presence of exposures such as aspirin and HRT use appear to modify relative risk associated with other the important colon cancer relative risk factors such as obesity. Therefore, changes in prevalence of aspirin and HRT use over time will affect colon cancer relative risk estimates associated with these other factors. The result of multiple risk factors changing overtime further illustrate the complexity of multi-factorial diseases, such as colon cancer, both in terms of risk factor identification and in understanding the disease process itself.

As documented here and by others, important diet and lifestyle factors that are associated with colorectal cancer have changed over time [16,30-33]. Obesity has been documented as increasing worldwide [34]. In the study by Cronin and colleagues[16], changes in five colon cancer risk factors, vegetable intake, red meat intake, physical activity, weight, and alcohol, were evaluated over a 20-year time period. Although they noted that changes had occurred in these factors over time, they summarized that the overall effect on colon cancer incidence associated with these changes was minimal since changes in risk and protective factors offset each other. They did not evaluate the impact of effect-measure modification when factors such as NSAIDs that change over time modify the observed relative risk associated with other exposures. Looking at a similar time period as we did in this study, Li and colleagues evaluated changes in diet, physical activity, and weight between 1990 and 1996 [35]. They observed that increases in fruit and vegetable intake were restricted to those who were active and had a normal weight, suggesting that differential shifts in the population are occurring.

Few studies have reported on changes in aspirin or HRT use over time and different definitions of regular use make comparisons across studies difficult. One of the first studies to detect an association between aspirin use and colon cancer was conducted by Rosenberg and colleagues who reported 7% of the population using aspirin in the late 1970s [26]. In 1976 the National Health Survey observed that roughly 20% of the population used any aspirin [36]. A survey conducted in Minnesota in 1981–82 indicated that among those between 55 and 74 years 14.6% of women and 13.6% of men took aspirin regularly (at least 7 of the prior 14 days), and that this increased to 19.8 % and 19.4% by 1985–86 [37]. That study noted that an increase in the proportion of study participants who reported use of aspirin for cardiovascular disease protection accounted for much of the change. A study of nurses reported that 11.6% of those younger than 52 years reported using NSAIDs over 3 times a week in 1997 and 18.8% and 27.9% of nurses over 52 years reported NSAID and aspirin use over 3 times per week [29,38]. In the late 1990s, NSAID use 3 or more times per week for at least one month was reported by 42% of our study population. Although difficulties in monitoring trends in aspirin use exist, taken together these reports are consistent with an upward trend in use over this time period.

While our study data indicate that HRT use increased during the 1990s, recent research findings regarding potential adverse effects from HRT use [39] are resulting in a dramatic reduction in the proportion of post-menopausal women using HRT[40]. Estrogen and HRT also have been identified as important modifiers of other risk factors, such as obesity, and may explain many of the differences in colon cancer obesity associations observed for men and women [10,11].

There are several strengths to the study, including a large sample to look at interactions to test our hypothesis, and the ability to look at data collected in the same manner from two population-based control groups, to identify exposure changes overtime. Response rates were slightly lower for the rectal cancer study than for the colon cancer study, although good response rates overall were obtained in both studies. However, because the study in the early 1990s was one of colon cancer and the one in the late 1990s was of rectal, we are unable to directly calculate relative risk estimates for the two time periods at the population level.

These findings also have important study design and analysis implications because they suggest that disease associations are complicated when factors that modify risk change. Aspirin, NSAID, and HRT use are examples of these types of effect-measure modifiers. Our results suggest that the risks associated with obesity and HRT is influenced by NSAIDs. In this scenario, case-control studies provide a snapshot of what risk factors are in the population at a given point in time. Studies with sufficiently large sample size can define subsets of the population that have different characteristics. Cohort studies accumulate cases over time; in order to take into account changing population characteristics, changes in risk factors for should be taken into account by incorporating repeated exposure measurements. The importance of identification of effect-measure modification should be stressed since results can have implications for understanding the data and the disease process. Often evaluation of effect-measure modification is secondary and stratified estimates are not presented unless significant effect-measure modification is detected. However, studies are often underpowered to detect statistically significant effect-measure modification [41]. When effect-measure modification between two risk factors has been reported, stratified odds ratios should be reported so that results can be compared between studies. Failure to evaluate effect-measure modification when knowledge of biological mechanisms, or prior data, suggest that it exists may lead to inability to recognize and understand associations that are important.

In summary, populations are dynamic and risk factors exist in a shifting context. Of particular importance are factors that modify the risk of other factors. NSAID use, HRT, and obesity are three factors that appear to be changing over time as well as having an impact as effect-measure modifiers of other diet and lifestyle factors. The impact of population changes on the epidemiologic literature can be kept to a minimum if investigators assess exposure-disease associations within strata of other exposures, and present results in a manner that allows comparisons across studies.

## Supplementary Material

Additional file 1 Numbers used to estimate population risk shown in Figure 2. The data provide a guide to calculations made for Figure 2 Click here for file
