# Supplementary material for: Changing population characteristics, effect-measure modification, and cancer risk factor identification
Source: Epidemiol Perspect Innov. 2007 Oct 1;4:10. doi: 10.1186/1742-5573-4-10 (PMC2098767; doi:10.1186/1742-5573-4-10)
Supplement: Additional file 1 — Numbers used to estimate population risk shown in Figure 2. The data provide a guide to calculations made for Figure 2 [file 1742-5573-4-10-S1.doc]

| Additional Files.  Numbers used to estimate population risk shown in Figure 2. | | | | |
| --- | --- | --- | --- | --- |
| Varying Percentages | | Aspirin/NSAID use | |  |
| Yes | No | OR Yes | OR No | weighted OR |
| 5 | 95 | 2.5 | 1.2 | 1.265 |
| 10 | 90 | 2.5 | 1.2 | 1.33 |
| 15 | 85 | 2.5 | 1.2 | 1.395 |
| 20 | 80 | 2.5 | 1.2 | 1.46 |
| 25 | 75 | 2.5 | 1.2 | 1.53 |
| 30 | 70 | 2.5 | 1.2 | 1.59 |
| 35 | 65 | 2.5 | 1.2 | 1.66 |
| 40 | 60 | 2.5 | 1.2 | 1.72 |
| 45 | 55 | 2.5 | 1.2 | 1.79 |
| 50 | 50 | 2.5 | 1.2 | 1.85 |
|  |  | HRT use | |  |
|  |  | OR Yes | OR No |  |
| 5 | 95 | 2.1 | 0.8 | 0.87 |
| 10 | 90 | 2.1 | 0.8 | 0.93 |
| 15 | 85 | 2.1 | 0.8 | 0.995 |
| 20 | 80 | 2.1 | 0.8 | 1.06 |
| 25 | 75 | 2.1 | 0.8 | 1.13 |
| 30 | 70 | 2.1 | 0.8 | 1.19 |
| 35 | 65 | 2.1 | 0.8 | 1.26 |
| 40 | 60 | 2.1 | 0.8 | 1.32 |
| 45 | 55 | 2.1 | 0.8 | 1.39 |
| 50 | 50 | 2.1 | 0.8 | 1.45 |
|  |  |  |  |  |
| Weighted OR based on % of population with use or yes have the OR | | | | |
| for yes and % of population with non-use or NO and OR associated with NO summed. | | | | |
